# A systematic review of the keystone design perforator island flap in lower extremity defects

**DOI:** 10.1097/MD.0000000000006842

**Published:** 2017-05-26

**Authors:** Jiuzuo Huang, Nanze Yu, Xiao Long, Xiaojun Wang

**Affiliations:** Division of Plastic and Reconstructive Surgery, Peking Union Medical College Hospital, Beijing, China.

**Keywords:** keystone design perforator island flap, keystone flap, lower extremity defect, perforator flap

## Abstract

**Background:**

The keystone design perforator island flap is useful for the reconstruction of lower extremity defects. We performed a systematic review with the objective of identifying complication rates associated with using the keystone design perforator island flap to treat such defects.

**Methods:**

The MEDLINE, PubMed Central, Embase, and Cochrane databases were searched from January 2003 to August 2016 for articles describing keystone design perforator island flaps in lower extremities. The study selection was performed in accordance with the Preferred Reporting Items for Systematic Reviews and Meta-Analyses statement.

**Results:**

Nine articles that involved a total of 282 keystone design perforator island flaps satisfied the inclusion criteria. In these articles, the most common cause of lower extremity defects was oncologic resection (89.0%). Most such defects were in the middle third of the lower leg (32.7%). Complications occurred in 9.6% of patients; these complications included partial flap loss (1.1%) and complete flap loss (0.7%).

**Conclusion:**

Given its high success rate and low technical complexity, if applicable, the keystone design perforator island flap should be the preferred approach for lower extremity reconstruction.

## 1 Introduction

The repair of lower extremity defects remains challenging for reconstructive surgeons due to a lack of suitable local tissue. Although free flaps are often utilized for larger defects, local perforator-based flaps may be ideal for smaller wounds that require coverage.^[1]^ Local perforator-pedicled propeller flaps can provide excellent form and function for both traumatic and atraumatic defects with minimal donor site morbidity but can have concerning rates of flap loss.^[2]^

As first described by Behan^[3]^ in 2003, the keystone design perforator island flap (hereafter referred to simply as the keystone flap) could be a useful solution with a high success rate for lower extremity defects.

To better place the keystone flap in the reconstructive decision algorithm, a systematic review of all published data in the literature was performed to identify rates of flap complications or failure for the keystone flap in lower extremity defects. Finally, recommendations for flap indications based on this review are presented.

## 2 Patients and methods

We followed the recommendations for interventional reviews provided in the Cochrane Handbook (version 5.1.0); our work was Assessment of Multiple Systematic Reviews (AMSTAR)-compliant, and our report was guided by the principles outlined in the Preferred Reporting Items for Systematic Reviews and Meta-Analysis (PRISMA) statement.^[4]^ Since it was a systematic review, it did not require ethical approval or patient consent.

### 2.1 Inclusion criteria

All the published original studies that described keystone flaps in lower extremity defects were included. Duplicate studies were excluded, as were review articles, purely technical descriptions, editorials, discussions, commentaries, and letters or viewpoints. For articles by the same author, we verified that data from different publications were not identical; any data that could possibly have been duplicated were excluded. For studies lacking full online data, we attempted to obtain access to complete data via direct request to the corresponding author. If multiple publications addressed the same study or portions of a study, we ensured that data from a single study were not counted repeatedly.

### 2.2 Search strategy

The MEDLINE, PubMed Central, Embase, and Cochrane Library electronic databases were searched from January 2003 (when the keystone flap technique was first described) to August 2016. This search was conducted using appropriate keywords in the English language combined with Boolean logical operators as follows: “keystone flap” OR “keystone design perforator island flap” [Title/Abstract/MeSH Terms]; “keystone flap” and “lower extremity” [Title/Abstract/MeSH Terms]. There were no limits on the search; if a foreign-language article was located, every effort was exerted to obtain an English copy or translate the article. Studies identified via electronic and manual searches were listed with their key information using Microsoft Excel 2011 (Microsoft Corp, Redmond, WA).

### 2.3 Data extraction and study appraisal

Data extraction was performed independently by 2 researchers (JH and NY), and disagreements were resolved by consensus. If consensus could not be achieved, 1 of the senior authors (XL) was asked to make the final decision. The following data were collected: age, location, and cause of the defect; complications; sample size; and follow-up. For each article, a level of evidence defined by the Oxford Centre for Evidence-Based Medicine was determined (Table 1).^[5–13]^

**Table 1:**
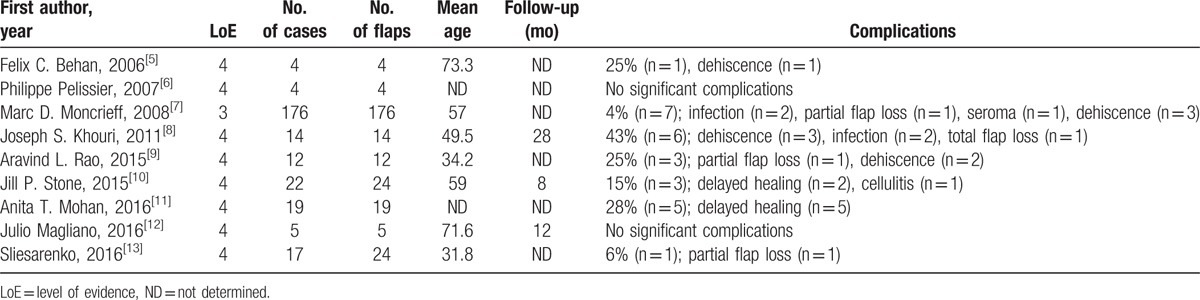
Synthesis of data from case studies and case series.

## 3 Results

### 3.1 Process outcomes

**Figure 1:**
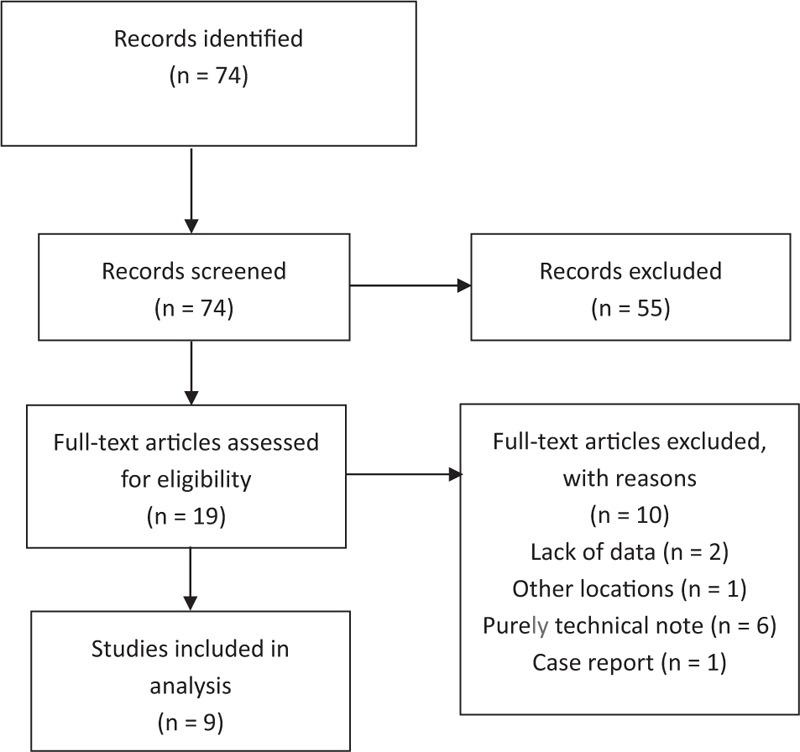
Flow diagram indicating the literature search and selection process, which were conducted in accordance with the Preferred Reporting Items for Systematic Reviews and Meta-Analysis statement.

A total of 74 references were identified via our search strategy, and we included 9 studies that satisfied our criteria for inclusion. Figure 1 presents the study selection process, including the identification, screening, and eligibility assessment steps.

The 9 articles involved 282 keystone flaps and 273 patients with lower extremity defects (Table 1). One article was a prospective cohort study;^[7]^ all other included articles were case series.^[5,6,8–13]^ The mean age of enrolled patients was 52.4 ± 6.4 years (range, 2–82 years), and the average follow-up duration was 15.3 ± 8.4 months.

### 3.2 Overview of practice

#### 3.2.1 Causes of defects

Causes (n = 236) were divided into 4 categories: oncologic resection (89.0%, n = 210), posttrauma (9.3%, n = 22), infection (0.9%, n = 2), and postoperative complications (0.9%, n = 2).

#### 3.2.2 Locations of defects

Most of the assessed defects (Table 2) involved the leg [n = 217 (88.6%)]. For defects in the leg, the most common defect location was the middle third of the lower leg [n = 80 (32.7%)], followed by the distal third of the lower leg [n = 72 (29.4%)].

**Table 2:**
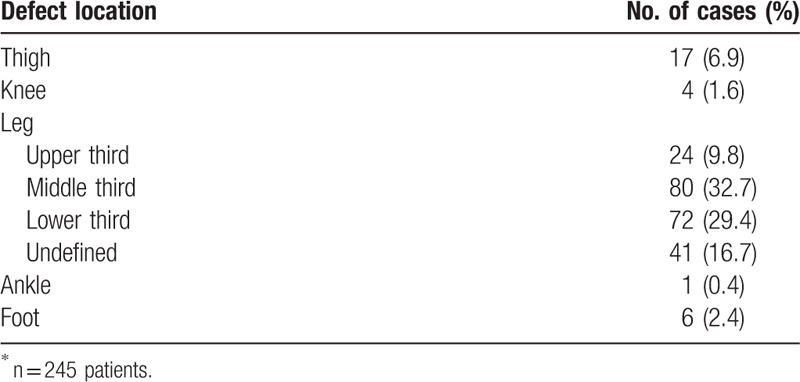
Locations of defects^∗^.

#### 3.2.3 Complications

Complications for all assessed flaps are identified below (Table 3). We found complications in 9.6% of cases (n = 27) and complete flap loss in 0.7% of cases (n = 2). The most frequent complications were wound dehiscence (5.7%, n = 16), infection (1.8%, n = 5), and partial flap loss (1.1%, n = 3).

**Table 3:**
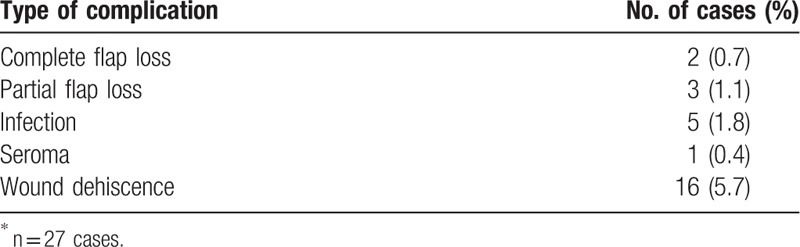
Types and incidences of complications^∗^.

## 4 Discussion

Lower extremity defects are challenging for reconstructive surgeons because of the lack of local tissue laxity. Due to this anatomical feature and frequent bone and/or tendon exposure, it is difficult for skin grafts to survive; as a result, many reconstructive surgeons consider free flaps as the first-line treatment option. In this context, muscular flaps have gradually been replaced by free perforator flaps with lower donor-site morbidity.

The term “perforator-based flap” was first used by Kroll and Rosenfield in 1988.^[14]^ Pedicled perforator flaps involve like-for-like tissue replacement, with the preservation of nerves and muscles and the main vascular trunks and reduced operating and hospitalization times.^[2]^

The most commonly applied pedicled perforator flap is the pedicled propeller flap. The propeller flap concept was first described in 1991 by Hyakusoku et al.^[15]^ In 2009, during the First Tokyo Meeting on Propeller Flaps, the propeller flap was defined as an island pedicled flap with an arc of rotation greater than 90°.^[16]^ As first described by Behan in 2003,^[3]^ the keystone flap, another local perforator-based flap, could be a useful solution for lower extremity defects. In contrast to a propeller flap, the keystone flap does not require meticulous dissection for the fine perforator or rotation of the thin perforators; thus, the use of the keystone flap can reduce operation time and decrease technical difficulty.^[12]^

Based on our literature search, 9 articles addressing the use of the keystone flap for lower extremity reconstruction were published during the preceding 10 years (2006–2016). The keystone flap was most often used after oncologic resection (88.98%, n = 210), followed by the reconstruction of posttraumatic defects (9.32%, n = 22), infection-related defects (0.85%, n = 2), and defects due to postoperative complications (0.85%, n = 2). The keystone flap could expand the armamentarium for dermatologic surgeons, plastic surgeons, and orthopedic surgeons.

There were 2 articles reporting more than 1 keystone flap on the same patient.^[10,13]^ Multiple keystone flaps on the same patient or on the same leg might affect the complication rate. Stone et al^[10]^ reported 32 keystone flaps in 30 patients, with 2 patients having bilateral lower leg flaps. However, there was no information on the complication about these 2 patients with bilateral lower leg flaps. Sliesarenko et al reported 24 keystone flaps in 17 patients. Among them, 5 patients had 2 keystone flaps, and 1 patient had 3 keystone flaps. However, none of those patients with multiple keystone flaps had any complication. Therefore, there was no enough information to conclude whether multiple keystone flaps on the same patient or on the same leg affect the complication rate.

The keystone flap procedure is safe and has low complication rates. We found complications in 9.6% of cases (n = 27) and complete flap loss in 0.7% of cases (n = 2). The most frequent complication was wound dehiscence (5.7%, n = 16), followed by infection (1.8%, n = 5) and partial flap loss (1.1%, n = 3). One published article compared complication rates for free and perforator-pedicled propeller flaps in lower extremity reconstruction.^[1]^ That article indicated that for free flaps and pedicled propeller flaps, complication rates were 19.0% and 21.4%, respectively, and complete flap loss rates were 3.93% and 2.77%, respectively. The complication rate and complete flap loss rate for the keystone flap are much lower than the corresponding rates for free flaps and pedicled propeller flaps.

Free flaps and pedicled propeller flaps require high levels of surgical experience. The use of pedicled perforator flaps avoids microanastomosis, which is time consuming and stressful for the surgeon.^[8]^ However, the pedicled propeller flap procedure remains complex, requiring the meticulous dissection of a thin perforator.^[17]^ In comparison, the keystone flap procedure is faster and more straightforward.^[3]^ Most of the authors of the included studies mentioned that keystone flaps required a short operating time,^[5,8–13]^ and 3 articles reported average operating time that was less than 2 hours (104,^[8]^ 45.5,^[9]^ and 68 minutes^[11]^). A literature review indicated that the average operating time for pedicled propeller flaps and free flaps is more than 2 hours.^[18–20]^ Since the keystone flap has a higher success rate and lower complexity than free flaps or pedicled propeller flaps, the keystone flap procedure should be the preferred procedure for lower extremity reconstruction.

However, the indications for the keystone flap are not the same as those for free flaps or pedicled propeller flaps. The keystone flap acts as a multiperforator advancement flap that requires local tissue laxity for advancement.^[12]^ If there is insufficient local tissue laxity for tissue advancement, the keystone flap cannot be applied for reconstruction. A pedicled propeller flap is an island-pedicled flap with an arc of rotation greater than 90°.^[16]^ With a known perforator and an arc of rotation, a pedicled propeller flap can repair a defect, as explained by the perforasome concept.^[21]^ A free flap remains the first-choice solution for covering large defects and for complex reconstruction requiring composite or functional reconstruction.^[2]^ Careful analysis should be performed prior to lower extremity reconstruction. If there is sufficient local tissue laxity for tissue advancement, the keystone flap could be used for reconstruction. Otherwise, a free flap or pedicled propeller flap should be utilized. However, there remains no consensus regarding selecting between a free flap and a pedicled propeller flap.^[1]^

Our study had several limitations. First, the reports that we reviewed featured different surgical techniques used by various surgeons. These techniques varied greatly and were thus far from standardized. Second, we lack data regarding comorbidities, defect locations, and donor site closure. Third, the included reports do not describe homogenous series of consecutive patients with lower-extremity wounds. Fourth, among the 9 articles included in this review, only 1 article described a prospective cohort study, whereas all of the remaining articles described case series; thus, the evidence provided by these studies was of relatively low strength. Ultimately, our analysis is observational in nature. A randomized controlled trial is desirable but will be difficult to perform because a large number of patients must be included to allow for the identification of significant between-flap differences.

## 5 Conclusions

The keystone flap has been a major step forward in the treatment of lower extremity defects during the preceding decade. Since the keystone flap has a higher success rate and lower complexity than free flaps or pedicled propeller flaps, if applicable, the keystone flap procedure should be the preferred approach for lower extremity reconstruction.
